# New indications and platforms for CAR‐T therapy in lymphomas beyond DLBCL

**DOI:** 10.1002/jha2.323

**Published:** 2021-11-23

**Authors:** Madiha Iqbal, Bipin N Savani, Mehdi Hamadani

**Affiliations:** ^1^ Division of Hematology and Oncology Mayo Clinic Jacksonville Florida; ^2^ Division of Hematology and Oncology Vanderbilt University Nashville Tennessee; ^3^ Blood & Marrow Transplantation and Cellular Therapy Program Division of Hematology and Oncology Medical College of Wisconsin Milwaukee Wisconsin

**Keywords:** allogeneic CAR, CAR‐T, dual CAR, follicular lymphoma, lymphoma, mantle cell

## Abstract

CD19‐directed chimeric antigen receptor T‐cell therapy (CAR‐T) represents a significant advancement for patients with relapsed/refractory large B‐cell lymphoma (LBCL). Long‐term follow‐up confirms durable remissions in nearly half of the patients, a population that was previously estimated to have a median survival of around 6 months with standard salvage therapy. This initial success of CAR‐T has led to significant expansion across other lymphoma histologies resulting in the recent regulatory approval of CAR‐T in mantle cell lymphoma and follicular lymphoma. Additionally, multiple novel platforms of CAR‐T therapy are under development to improve efficacy and limit toxicity such dual antigen targeting, allogeneic and natural killer CARs. In this review, we focus on the new indications of CAR‐T in lymphomas beyond LBCL as well as emerging platforms of CAR‐T therapy.

## 1 INTRODUCTION

Chimeric antigen receptor modified T‐cell (CAR‐T) therapy represents a novel treatment for patients with relapsed and/or refractory (R/R) large B‐cell lymphoma (LBCL). Axicabtagene ciloleucel (axi‐cel) followed shortly by tisagenlecleucel (tisa‐cel) were the first to receive Food and Drug Administration (FDA) and European Medicines Agency (EMA) approval for patients with R/R LBCL who had failed 2 or more lines of systemic therapy [1, 2, 3]. Long‐term follow‐up data confirms remissions lasting 3 years and beyond for approximately 40% of such patients after CAR‐T therapy [4]. Recently, lisocabtagene maraleucel (liso‐cel) was approved for LBCL in third or later line [5]. Objective response (OR) was achieved in 73% with complete remission (CR) in 53%. Notably grade ≥3 cytokine release syndrome (CRS) and neurological adverse events (NAEs) were seen in only 2% and 10% patients, respectively, [5].

The remarkable efficacy and manageable toxicity in a patient population that previously had dismal outcomes has not only led to significant expansion of CAR‐T across other disease subtypes, but it has also led to the development of new platforms of CAR‐T delivery. Mantle cell lymphoma (MCL) and follicular lymphoma (FL) are both heterogeneous B‐cell non‐Hodgkin lymphomas (NHL) that are considered incurable with standard chemoimmunotherapy (CIT) [6, 7]. CAR‐T with brexucabtagene (brexu‐cel) and axi‐cel is now commercially available for R/R MCL and FL respectively.<COMP: Please set Reference citations as per the journal style.>

In this review, we discuss new indications for CAR‐T in NHL beyond LBCL. We also discuss emerging experimental platforms of CAR‐T in lymphomas that are expected to challenge our current clinical practices.

## 2 MANTLE CELL LYMPHOMA

MCL comprises about 6% of all adult NHL and is generally aggressive in its clinical presentation although a subset present with an indolent clinical course [8, 9, 10]. Younger symptomatic, advanced disease patients are typically offered upfront intensive systemic CIT [6]. This is followed by a consolidative autologous hematopoietic cell transplant (auto‐HCT) and rituximab maintenance, although intensive CIT regimens without an auto‐HCT have also shown comparable outcomes [11, 12, 13]. Despite intensive upfront treatment, long‐term follow‐up confirms a continuous pattern of relapse with outcomes particularly poor for patients relapsing early after auto‐HCT [14, 15].

Significant progress has been made in MCL relapsing after front line CIT with the approval of multiple novel chemotherapy free treatments. Most notable are the Bruton's tyrosine kinase inhibitor inhibitors (BTKi) [16, 17, 18]. Despite initial high responses with BTKi the disease remains incurable with median progression‐free survival (PFS) of around 1 year [19]. Outcomes are particularly poor for patients relapsing after BTKi with a median overall survival (OS) of under 6 months, representing a strong clinical need for new treatments [20, 21].

ZUMA‐2 trial led to the approval of brexu‐cel, the first and currently the only approved CAR‐T for patients with R/R MCL. Brexu‐cel is a CD‐19‐directed auto‐CAR‐T with a CD3ζ signaling domain and a CD28 costimulatory domain [22]. In the pivotal trial 74 patients were enrolled, brexu‐cel was manufactured successfully for 71 (96%) and administered to 68 (92%) [22]. Included patients had previously received an anthracycline or bendamustine containing chemotherapy in combination with an anti‐CD20 monoclonal antibody (mAb) and a BTKi. Median age was 68 (range: 38–79), high‐risk prognostic features were common including blastoid morphology (*n* = 21, 31%), Ki67 ≥30% (*n* = 40, 82%) and *TP53* mutation (*n* = 6, 17%). Bridging therapy (BT) was administered to 25 patients (37%) [22]. Among the enrolled population, 85% had an OR with 59% achieving CR [22]. Notably, a positive correlation between expansion of CAR‐T and disease response was observed, consistent with prior studies [1]. The estimated PFS and OS at 12 months was at 61% and 83%, respectively [22]. Most common grade ≥3 adverse events (AEs) were cytopenias (94%) followed by infections (32%) [22]. CRS was reported in 91% with grade ≥3 CRS in 15% (Lee criteria) and NAEs were reported in 63% with grade ≥3 NAE in 31% (Table 1).

**TABLE 1:** Autologous CD19‐directed CAR‐T studies in mantle cell lymphoma and follicular lymphoma

CAR‐T Trial(ref no)	CAR‐T product/ construct	Patients enrolled (*n*)/CAR‐T infused (*n*)	Median age for patients receiving CAR‐T (range)	CR/ORR rate (%)	Grade ≥ 3 CRS/ICANS (%)
**Mantle cell lymphoma**					
ZUMA‐2 [22]	Brexu‐cel/CD28 costimulatory domain	74/68	65 (38–79) years	59/85^a^	15/31
TRANSCEND‐NHL‐001‐MCL cohort [23]	Liso‐cel/4‐1BB costimulatory domain	40^b^/32	67 (30–80) years	59/84	3/9

**Follicular lymphoma**					
ZUMA‐5 [36]	Axi‐cel/CD28 costimulatory domain	124 infused (data reported for 84)	61 (34–79) years	80/94	6/15
ELARA [39]	Tisa‐cel/4‐1BB costimulatory domain	98/97	57 (29–73)	65/83^c^	0/2

Abbreviations: CAR‐T, chimeric antigen receptor T cell; Brexu‐cel, brexucabtagene, Liso‐cel, lisocabtagene maraleucel; Axi‐cel, Axicabtagene ciloleucel; Tisa‐cel, tisagenlecleucel; CR, complete remission; ORR, overall response rate; CRS, cytokine release syndrome; ICANS, immune effector cell‐associated neurotoxicity syndrome.

^a^ For all enrolled patients.

^b^ Underwent leukapheresis.

^c^ Reported for 52 patients evaluable for efficacy.

Liso‐cel is another CD19‐directed CAR‐T with a CD3ζ signaling domain and a 4‐1BB costimulatory domain [5]. During manufacturing of liso‐cel, CD4+ and CD8+ T cells are separated from the leukapheresis product and thereafter individually activated, expanded, and administered as two separate sequential infusions of equal doses [5]. Preliminary results on the safety and efficacy of liso‐cel in R/R MCL were reported at the American Society of Hematology (ASH) annual meeting, 2020 [23]. Forty patients underwent leukapheresis and liso‐cel was administered at dose level (DL) of 50 × 10^6^ CAR T cells (*n* = 6) or 100 × 10^6^ CAR T cells (*n* = 26) to 32 patients [23]. Median patient age was 67 years (range: 36–80) [23]. High‐risk disease features such as blastoid morphology, high Ki67 index, *TP53* mutation and complex karyotype were reported in 37.5%, 72%, 22%, and 34% of patients, respectively [23]. Twenty‐eight (87.5%) had received prior BTKi and 11 (34%) were assessed to be refractory to BTKi [23]. BT was administered to 17 patients (53%) [23]. Twenty‐seven (84%) had grade ≥ 3 AEs, most common being neutropenia followed by anemia and thrombocytopenia [23]. CRS was observed in 16 (50%) with grade ≥3 CRS in only one patient. NAEs were observed in 9 (28%); 3 patients experienced grade ≥3 NAE [23]. OR was observed in 27 patients (84%) with CR in 19 (59%) (Table 1).

It is important to note that in ZUMA‐2 all enrolled patients had prior BTKi therapy; however, the FDA approval is broad and allows any patient with R/R MCL to be eligible for CAR‐T, regardless of prior receipt of BTKi. This is different from the regulatory approval from the EMA, which is restricted to patients who have had prior BTKi. How to best sequence the currently available treatment options in patients with R/R MCL is largely unknown. In spite of outstanding responses with CAR‐T in patients with R/R MCL, long‐term follow‐up is much awaited, and the toxicities and costs associated with CAR‐T are not negligible [24]. The recently published guidelines from American Society of Transplantation and Cellular Therapy, Center of International Blood and Marrow Transplant Research, and European Society for Blood and Marrow Transplantation recommend CAR‐T in MCL for patients who are intolerant to or relapse after at least one BTKi [25]. Notable exception is only *TP53* mutated R/R MCL where an earlier receipt of CAR‐T, prior to BTK exposure may be reasonable [19, 25].

## 3 FOLLICULAR LYMPHOMA

FL is the most common indolent NHL comprising about 35% of all adult NHL [26]. The rituximab era has seen significant improvement in long‐term outcomes for patients with FL with 10‐year OS of ∼80% [27]. Despite excellent long‐term outcomes, FL remains a remarkably heterogenous histology. Various clinical, biological, and genetic prognostic models have been proposed to understand the inherent heterogeneity of FL such as FL international prognostic index (FLIPI), m7‐FLIPI, and progression within 2 years of front line CIT (POD24) [28, 29, 30]. The treatment options in R/R FL are fairly diverse with no single treatment modality shown to be superior and range from CIT, radioimmunotherapy, immunomodulators, and most recently, novel agents such as the PI3K inhibitors and tazemetostat, an EZH2 inhibitor [7, 31–33]. Auto‐HCT and allogeneic (allo) HCT have been both investigated in R/R FL; however, the exact role of each remains largely controversial [34, 35].

Axi‐cel was approved in the United States in 2021 for the treatment of patients with R/R FL after at least two lines of systemic therapy. The approval was based on the primary analysis of ZUMA‐5 trial [36]. One hundred and twenty‐four patients with grade 1 to 3a FL or marginal zone lymphoma (MZL) (*n* = 22) who had previously received two lines of therapy received axi‐cel [36]. Axi‐cel was infused at a dose of 2 × 10^6^ cells. Median age was 61 years (range: 34–79) with 57% being male [36]. All patients were heavily pretreated; adverse prognostic features were reported in nearly half of the patients with ECOG >1, stage III/IV disease, ≥3 FLIPI, high tumor bulk and POD24 in 62%, 86%, 47%, 49%, and 55%, respectively [36]. Safety and efficacy data were reported for 84 FL patients with at least 12 months follow‐up. OR was 94% with a CR in 80%. For patients with MZL, OR was 85% with CR in 60%. Most common grade ≥3 AE was neutropenia followed by anemia [36]. Grade ≥3 CRS and NAE were reported in 6% and 15% of patients with FL. A higher incidence of Grade ≥3 NAE was reported in patients MZL at 41% [36]. Outcomes were also reported for nine patients who received retreatment with axi‐cel upon disease relapse [37]. These patients had disease relapse at 3‐month post infusion after initially achieving a OR and maintained CD19 expression at relapse [37]. All patients showed evidence of OR to retreatment, and safety profile was not different from first infusion [37]. Updated outcomes for these patients and two additional patients with FL were recently reported and median DOR remains not reached at 11.4 months [38] (Table 1).

Tisa‐cel has also shown efficacy and safety in R/R FL based on the planned interim analysis of ELARA trial [39]. Patients with grade 1 to 3a FL who had disease relapse within 6 months of second line or later CIT or had disease relapse post auto‐HCT were included. Tisa‐cel was infused at a dose of 0.6–6 × 10^8^ CAR‐T to 97 patients. Median age was 57 years (range: 29–73); 66% were male, 84% had advanced stage disease, and 60% had FLIPI score ≥3. Thirty‐six percent had prior auto‐HCT, 77% had refractory disease to last therapy, and 60% had POD24. Forty‐three percent received BT and 18% received tisa‐cel in the outpatient setting. Fifty‐two patients were assessed for efficacy and had a median follow‐up of 9.9 months. Forty‐three patients (83%) had an OR with 34 (65%) achieving a CR. Responses were seen across all disease prognostic subgroups. Median DOR, PFS, and OS were not reached at last follow‐up. Most common grade ≥3 AE was neutropenia. CRS was reported in 48%; maximum CRS grade was 2. NAEs were reported in 10%; 2% experienced grade ≥3 NAE. No treatment related death was reported (Table 1).

As noted, there are multiple treatment options available today for patients with R/R FL; however, short of an allo‐HCT, none of the treatment options are curative [40]. Despite the increase in treatment options, long‐term outcomes for patients decline sharply after second line of therapy with continued decrease in PFS and OS with each subsequent line [41, 42]. Recently, a comparison of ZUMA‐5 with SCHOLAR‐5 was presented at the European Hematology Association Meeting [43]. SCHOLAR‐5 is a retrospective external control cohort of R/R FL patients who had initiated 3rd or higher line of therapy after July, 2014. Eighty‐six patients from ZUMA‐5 and 85 from SCHOLAR‐5 were included with median follow‐up of 23.3 and 26.2 months, respectively; both cohorts were balanced through propensity scoring. Baseline characteristics were similar between the two cohorts except performance status; ZUMA‐5 had a higher number of patients with poor performance scores [43]. OR, CR, PFS, and OS favored ZUMA‐5 over SCHOLAR‐5. Similar trend was observed when patients who had received four or more lines of therapy were compared [43]. These data support the use of axi‐cel in patients who have received at least two lines of prior systemic therapy, consistent with the current regulatory approval.

## 4 EXPERIMENTAL AUTOLOGOUS CAR‐T PLATFORMS

CD‐19‐directed auto‐CAR‐T represents a significant milestone in the treatment of patients with R/R NHL. However, disease relapse remains a significant hurdle with long‐term durable responses seen in only about 40–50% of patients [44]. Various mechanisms have been elucidated regarding failure of CAR‐T including antigen loss, host immune dysregulation, and exhausted T‐cell repertoire [1, 45, 46]. Here we discuss targets beyond CD19 and new auto‐CAR‐T platforms that are being investigated in lymphomas with promising early results.

### 4.1 CD30‐directed CAR‐T

CD30 represents a viable target for CAR‐T in Hodgkin lymphoma (HL) as it is uniformly expressed on malignant Hodgkin and Reed‐Sternberg cells and has limited expression on normal tissue. CD30 is a member of the tumor necrosis factor superfamily; signal transduction via CD30 activates NF‐kB, enhancing apoptosis of malignant cells [47, 48]. Recently the results of two parallel conducted Phase I/II trials of CAR‐T targeting CD30 in R/R HL were published [49]. Outcomes were reported for 42 adult patients who had progressed after at least two lines of therapy. Median age of treated patients was 35 years (range: 17–69) with 7 median prior lines of therapy (range: 2–23) [49]. Safety profile was excellent with no NAE reported and a maximum of grade 1 CRS in 10 patients (24%) [49]. Thirty‐seven were evaluable for response; OR was achieved in 23 (62%) with CR in 19 (51%). Three different LD regimens were employed, namely bendamustine alone, fludarabine in combination with bendamustine, and fludarabine in combination with cyclophosphamide. Fludarabine‐based LD chemotherapy regimens were associated with the highest response. At a median follow‐up of 533 days, 1‐year PFS and OS was at 36% and 94%, respectively [49], raising concerns about durable disease control.

CD30 targeting CAR‐T holds promise for lymphomas beyond HL. Early trials have shown safety and encouraging responses in patients with CD30 expressing R/R anaplastic large cell lymphomas [50, 51] (Table 2).

**TABLE 2:** Experimental Autologous CAR‐T platforms in clinical trials

CAR‐T Trial (ref no)	Target antigen/construct	Histology/CAR‐T infused (*N*)	Median age of patients receiving CAR‐T (range)	Median number of prior therapies (range)	CR/ORR rate (%)	Grade ≥ 3 CRS/ICANS (%)
**Single antigen targeting CAR‐T**						
Baylor College of Medicine and University of North Carolina [49]	CD30/ Retroviral vector; CD28 costimulatory domain	HL/42	35 (17–69) years	7 (2–23)	51/62^a^	0/0
National institute of Health/Stanford University [53]	CD22/4‐1BB costimulatory domain	B‐cell ALL, LBCL/58	17.5 (4.4–30.6) years	N/A	70	10/2
Legend Biotech LB1901/ Shanghai Jiao Tong University School of Medicine Anti‐TRBC1 [61, 62]	CD4/ Lentiviral vector; 4‐1BB costimulatory domain/ TRBC1	LB1901: PTCL/AITL/CTCL Anti TRBC1: PTCL/AITL/T‐cell ALL/ALCL	N/A	N/A	N/A	N/A
Baylor College of Medicine; Houston Methodist Hospital [60]	CD5/ Retroviral vector; CD28 costimulatory domain	T‐cell ALL, T‐cell NHL/9	62 (16–71) years	5 (2–18)	33/44	0
**Dual antigen targeting CAR‐T**						
Medical College of Wisconsin [54]	CD19 and CD 20/4‐1BB costimulatory domain	B‐cell NHL, CLL/22	57 (38–72) years	4 (2–12)	64/82	5/14
Stanford University [55]	CD19 and CD22/Lentiviral vector; 4‐1BB costimulatory domain	B‐cell ALL, LBCL/ 21^b^	70 (25–78)^b^ years	3 (2–7)^b^	29/62^b^	5/5^b^
Alexander Study of AUTO3 [56]	CD19 and CD22/ Retroviral vector; 4‐1BB costimulatory domain	LBCL/33	59 (28–83)	3 (1–10)	52/69^c^	0/9

Abbreviations: CAR‐T, chimeric antigen receptor T‐cell; HL, Hodgkin lymphoma; NHL, non‐Hodgkin lymphoma; CLL, chronic lymphocytic leukemia; B‐cell ALL, acute lymphoblastic leukemia; LBCL, large B‐cell lymphoma; PTCL, peripheral T‐cell lymphoma; AITL, angioimmunoblastic T‐cell lymphoma; CTCL, cutaneous T‐cell lymphoma; ALCL, anaplastic large‐cell lymphoma; CR, complete remission; ORR, overall response rate CRS, cytokine release syndrome; ICANS, immune effector cell‐associated neurotoxicity syndrome

^a^ Evaluable patients (*n* = 37).

^b^ LBCL patients only.

^c^ Evaluable patients (*n* = 29).

### 4.2 CD22‐directed CAR‐T

CD22 represents another target for CAR‐T in patients with B‐cell malignancies as it is expressed exclusively on malignant B cells [52]. The results of a Phase I dose escalation study of anti‐CD22 CAR‐T in R/R CD22+ B‐cell malignancies were recently reported [53]. Fifty‐eight patients received anti‐CD22 CAR‐T; 51 (87.9%) had prior anti‐CD19 CAR‐T [53]. Among treated patients, one had diffuse large B‐cell lymphoma. CAR‐T cell dose level (DL) ranged from 3 × 10^5^/kg to 3 × 10^6^/kg [53]. Increased toxicity, specifically hemophagocytic lymphohistiocytosis was observed in 19 (32.8%) after CD4/CD8 T‐cell selection was incorporated [53]. A lower dose of 3 × 10^5^/kg was selected for dose expansion thereafter. CRS was overall reported in 50 patients (86.2%) and ranged from grade 1 to 2 in 45 (90%). NAE were reported in 19 (32.8%) with severe NAE in only 1. Forty patients (70.2%) achieved CR [53] (Table 2).

### 4.3 Dual antigen targeting in lymphoma

Traditional CARs are directed against a single tumor antigen (e.g., CD19) and their use has been associated with antigen negative (e.g., CD19–) relapses. CARs targeting more than one tumor antigen theoretically may have improved efficacy and/or lower probability of antigen negative disease at release. Investigators at the Medical College of Wisconsin conducted a first‐in‐human trial of bispecific anti‐CD20, anti‐CD19 CAR‐T for adult patients with B‐cell NHL or chronic lymphocytic leukemia (CLL) [54]. The study used on‐site manufacturing using the CliniMACS Prodigy system. CAR‐T cell dose ranged from 2.5 × 10^5^ to 2.5 × 10^6^ cells/kg. Grade ≥3 CRS occurred in one (5%) patient, and grade ≥3 NAEs occurred in three (14%) patients. Eighteen (82%) patients achieved an OR at day 28, including 14 (64%) CR. Notably, loss of the CD19 antigen was not seen in patients who relapsed [54] (Table 2).

Early results from two Phase I trials with bispecific anti‐CD22, anti‐19 CAR‐T in LBCL have also been encouraging [55, 56]. Patients with CD19+ LBCL who had at least two lines of prior therapy received bispecific anti‐CD19, anti‐CD22 CAR‐T (*n* = 21); no patient had prior receipt of CD19 CAR‐T. CAR T‐cell DL ranged from 1 × 10^6^/kg to 3 × 10^6^ cells/kg [55]. Grade ≥3 CRS and NAE occurred in one patient each. Best OR at any time was 62% (*n* = 13) with CR in 29% (*n* = 6) [55]. Interestingly, 29% (*n* = 4) patients relapsed with CD19 negative disease but retained expression of CD22 [55]. Sequential infusion of anti‐CD22 and anti‐CD19 CAR‐T is another strategy for dual antigen targeting that has shown encouraging responses. Of note no patient had antigen negative disease relapse in this study (Table 2) [57].

### 4.4 Targeting T‐cell antigens

T‐cell lymphomas (TCL) represent a biologically heterogeneous group of lymphomas, typically having an aggressive disease presentation. However, development of CAR‐T in TCL in comparison to their B‐cell counterparts is challenging due to antigen sharing between malignant T cells and CAR‐T; this can lead to a higher risk of antigen masking, fratricide, and T‐cell aplasia [58, 59]. Targeting CD5 as it is a pan T‐cell marker has been evaluated with modest results and other strategies are underway[60]. CD4 is uniformly expressed on most T‐cell lymphomas and represents a target for CAR‐T in TCL. LB1901 is an anti‐CD4 targeting CAR‐T construct that has shown strong antitumor effects in in vivo and in vitro models with no evidence of antigen masking [61]. A Phase I, first‐in‐human trial of LB1901 in adult patients with R/R CD4+ TCL is registered and is about to start recruitment (NCT04712864). T‐cell aplasia can be limited by targeting T‐cell receptor (TCR) β‐chain constant domain 1 and 2 (TRBC1 and TRBC2) as malignant T‐cell express TRBC1 or TRBC2 exclusively unlike their normal counterparts [62]. In preclinical models, anti‐TRBC1 CAR‐T showed antitumor efficacy while sparing normal T cells expressing TRBC2 [62]. A Phase I trial of anti‐TRBC1 CAR‐T in patients with R/R TRBC1 expressing TCL is currently recruiting (NCT04828174) (Table 2).

## 5 LIMITATIONS OF AUTOLOGOUS CARS

Despite impressive activity in B‐cell lymphomas and commercial availability, the auto‐CAR‐T construct suffers from several practical limitations (Table 3). First, reliable manufacturing and rapid access are key requirements for the broader application of cellular therapies. Unfortunately, auto‐CAR‐T treatments require a time intensive bespoke manufacturing process. In the pivotal B‐cell lymphoma CAR‐T trials the median turnaround time from apheresis to infusion of CAR product ranged from 15 to 54 days and CAR‐T manufacturing failure was reported in 1–8% of the intended recipients [1, 63–65]. Second, the product T‐cell composition, fitness, and expansion kinetics are important determinants of anti‐CAR responses [67–69]. For example, CAR‐T expansion during manufacturing [69] and enrichment of the final product with central or stem cell memory phenotype have been shown to correlate with efficacy outcomes [68]. These factors in turn are partly dependent of patient factors and prior treatments, leading to significant variability in the infused product characteristics across patients. Third, disease relapse remains a significant clinical problem. Among patients with DLBCL, >50% of patients relapse within a year of receiving CAR‐T [1, 2, 22]. Finally, the cost of commercially available products is high and poses a significant barrier to their widespread [70]. Cost‐effectiveness analyses suggest that compared to chemotherapy, the incremental cost‐effectiveness ratio (ICER) of CAR‐T therapies for DLBCL is a modest $136,000 per quality‐adjusted life year (QALY) gained [71]. Modifications in the manufacturing technology, for examples, decentralized model of CAR‐T production [72] or use of off‐the‐shelf CAR‐T products may mitigate the costs compared to the current model.

**TABLE 3:** Potential advantages and disadvantages of autologous and allogeneic CAR platforms

**Autologous CAR‐T**	**Allogeneic CAR‐T**
**Advantages**	**Advantages**
Commercially available for some lymphoma sub types (e.g., aggressive B‐cell lymphoma, mantle cell lymphoma, follicular lymphoma) Toxicity profile known No risk of GVHD or immunologically mediated rejection	Off‐the‐self (potential to treat all eligible patients) Repeated dosing maybe feasible No need for apheresis and associated logistical delays Standardization of T‐cell phenotype and fitness maybe possible with less product variability (e.g., CAR‐T phenotype, exhaustion)
**Disadvantages**	**Disadvantages**
High cost Logistical challenges (for collection/shipping; interval between leukapheresis to CAR‐T administration) Manufacturing failure Variable T‐cell fitness and composition Retreatment typically not feasible	Risk of GVHD Rejection risk Unknown persistence potential Insertional mutagenesis Profound immunosuppression and risk of infections (with some platforms) Maybe limited by healthy donor pool availability Commercial scalability and production remain to be proven Unknown long‐term safety

Abbreviations: CAR, chimeric antigen receptor; GVHD, Graft‐versus‐Host Disease.

## 6 PROMISE OF ALLOGENEIC CAR CONSTRUCTS

Allo‐CARs (derived from healthy donors or stored cellular products) as a potential “off‐the‐shelf” treatment may circumvent some of limitations associated with auto‐CARs (Table 3). If allo‐CARs live up to their potential of being readily available cellular therapy products, they may obviate the need for bridging treatments and address manufacturing failure occasionally seen with autologous platforms. Whether donor pool, scaling, and manufacturing process would be efficient enough to meet demand remains to be seen. Theoretically allo products can have less variability in terms of T‐cell composition and fitness, but available data to confirm this are not available. These products are also touted as cost friendly options, but this remains unknown at this point.

## 7 ALLOGENEIC CARS’ POSSIBLE PITFALLS

Before the potential benefits of allo‐CAR‐T therapies are clinically realized, potential pitfalls associated with approach need close attention. The main barrier for universal CAR‐T products is alloreactivity, which results from the donor–recipient human leukocyte antigen (HLA) disparity imparting a bidirectional risk, that is, to the cellular product (from the recipient immune system) and to the recipient in vivo (from the CAR‐T). This alloreactivity when mediated by the recipient T and NK cells can lead to the rejection of allo‐CARs, thereby limiting the anticancer efficacy. As with conventional unrelated donor allo‐HCT, preexisting antibodies, called donor‐specific anti‐HLA antibodies (DSA), can also mediate immune rejection if the host has been previously sensitized against HLA antigens (e.g., by multiple transfusions, pregnancies) [73] and therefore, screening for DSA in the recipient may be a necessary step before allo‐CAR administration. Several strategies are under investigation to minimize the risk of allo‐CAR rejection. Suppression of HLA class I expression by disrupting the *HLA‐A* or β2‐microglobulin (B2M; nonpolymorphic subunit of HLA‐I complex) genes in allo‐CAR‐T via gene editing would allow T cells to evade elimination by the host immune system [75–79]. Knocking out B2M reduces surface expression of HLA class I; however, these HLA‐I negative universal T cells could still be rejected by recipient NK cells [79]. Employing an anti‐NK‐cell depleting antibody or engineering T cells with HLA‐E expression are possible solutions to evade NK‐mediated rejection [80, 81].

In the other direction, the allo‐CAR‐T reactivity directed against the host can lead to the development of lethal graft‐versus‐host disease (GVHD) [82]. One strategy to reduce the risk of GVHD is the use of allo‐virus‐specific T cells (VST) CARs. The administration of such allo‐T cells with a narrow TCR repertoire may have a lower risk of initiating GVHD [83, 84, 85, 86]. Another approach is to disrupt the native TCR through deletion of TCR α constant (TCRAC) or TCRBC genes in the allo‐T cells, using gene‐editing technologies [74, 75, 76]. CAR‐T lacking surface TCR expression are incapable of mounting an alloreactive response against the recipient. However, depending on the gene editing method used, some unedited, TCR‐bearing T cells may remain and can potentially cause GVHD.

Expansion and persistence of CAR cells are vital to achieve short‐term control and may be important for long‐term efficacy in certain diseases [87]. The risk of alloimmunization is also a concern, where new CAR‐specific antibody generation in the recipient may limit redosing of the allo‐CAR‐T [88]. Disrupting HLA expression alone may not be sufficient for CAR‐T long‐term persistence and efficacy. Further manipulations of the T cells may be needed, for example, knocking out T‐cell inhibitory receptors such as PD‐1, TIM‐3, LAG‐3, and CTLA‐4 can enable CAR‐T to avoid exhaustion, improve persistence, and evade immunologic response [79, 89, 90]. Allo‐CAR‐NK are limited by their short lifespan of a few weeks without cytokine support; however, recent advances such as incorporation of cytokine transgenes (e.g., interleukin [IL]‐2 or IL‐15) can enhance NK‐cell proliferation and survival [91].

With genomic editing technologies, oncogenesis via oncogene activation or disruption of tumor suppressor genes is potential concerns [92]. It is critical to avoid insertional oncogenesis by using approaches such as optimized sgRNA design and Cas9 activity, prior off‐target detection assays, and careful selection of target loci [93, 94]. Bishop et al. generated anti‐CD19 CAR‐T from HLA‐identical sibling donors of allo‐transplant recipients with relapsed B‐cell acute leukemia or high‐grade NHL, using the high‐capacity piggyBac transposon, instead of a viral vector [95]. Two patients in the cohort of 10 developed CD19+ T‐cell malignancies, raising questions about the oncogenic potential of the piggyBac system.

## 8 EXPERIMENTAL ALLOGENEIC CAR‐T PLATFORMS

Several allo‐cellular therapy platforms are being actively investigated in lymphoid malignancies (Table 4). Most allo‐T‐cell therapies have used αβ T cells with knocked out TCR to eliminate alloreactivity. Other platforms have restricted or invariant TCRs, like γδ T cells, VSTs, or natural killer (NK) cells.

**TABLE 4:** Experimental allogeneic CAR‐T platforms in clinical trials

CAR‐T trial (ref no)	Platform	Histology; *N*	CR/ORR rate (%)	Grade ≥ 3 CRS/ICANS (%)	Comments
NK cells					
MD Anderson Cancer Center [98]	Cord blood antiCD19 NK‐cell CAR, with IL‐15 gene to enhance persistence and inducible caspase 9 kill switch	NHL; *N* = 11	63/72	0/0	No cases of GVHD
FT516 (Fate Therapeutics) [99]	Clonal master iPSC line engineered with NK CAR that targets CD19; a novel high‐affinity 158V, noncleavable CD16 Fc receptor, and an IL‐15 receptor fusion	NHL; *N* = 11	73/55	NA	
**Alpha‐beta T cells**					
Allogene 501 [101, 102]	TALEN‐mediated CD52 & TRAC knock out; rituximab recognition domain as kill switch, CD19 CAR	DLBCL or FL; *N* = 32	50/75	0/2	No reports of GVHD. Better CAR persistence in patients achieving CR
PBCAR0191 (Precision Biosciences) [106]	Propriety gene‐editing platform disrupting TCR by CD19 CAR insertion into the TRAC locus	NHL; *N* = 16	38/69 at day 28	0/0	Higher responses and toxicity with escalated doses of lymphodepletion
CARBON (CTX110) [112]	CRISPR/Cas9‐editing to disrupt endogenous TCR and β_2_‐microglobulin to eliminates HLA class I expression	DLBCL or FL‐3b; *N* = 0	NA	NA	Trial actively enrolling
TT11X (EBVSTs) [113]	CD30 CAR in EBV specific T cells	CD30+ lymphoma; *N* = 6	60%	NA	Trial actively enrolling

**Gamma‐delta T cells**					
Adicet Bio	Gamma‐delta T‐cell‐derived CD20 CAR	NHL; *N* = 0	N/A	N/A	Trial starting enrollment

Abbreviations: CAR, chimeric antigen receptor; CR, complete remission; CRS, cytokine release syndrome; DLBCL, diffuse large B‐cell lymphoma; FL, follicular lymphoma; GVHD, Graft‐versus‐Host Disease; NHL, non‐Hodgkin lymphoma; NK, natural killer; ICANS, immune effector cell‐associated neurotoxicity syndrome; ORR, overall response rate.

### 8.1 Allogeneic NK‐cell CARs

NK cells play a pivotal role in immune surveillance by targeting cancer or virally infected cells that down regulate HLA class I molecules [96]. Allo‐NK cells have been used for adoptive immunotherapy for cancer patients with excellent safety profile [97], and now NK‐cell‐derived CARs are being investigated in B‐cell lymphomas and other malignancies. The group at MD Anderson Cancer Center pioneered the use of CD19‐directed NK‐CARs derived from umbilical cord blood (UCB) units [98]. The study used a retroviral vector carrying genes that encoded CD19‐directed CAR, IL‐15 to enhance the in vivo expansion and persistence of the transduced NK cells (“Armored CAR”), and an inducible caspase 9 to trigger apoptosis of the CAR‐NK (as a safety switch). The results showed a promising safety and efficacy profile in 11 NHL and CLL patients with clinical responses observed in 73% of patients [98] (Table 4).

Other NK‐cell source beyond UCB includes induced pluripotent stem cells (iPSCs) and NK‐cell lines. FT516 is a CD19‐specific NK‐CAR in development against relapsed, refractory B‐cell NHL, and is engineered from a clonal master iPSC line, with clustered regularly interspaced short palindromic repeats (CRISPR)‐mediated insertion of the CAR at the TRAC locus (Table 4). The key attributes include a proprietary CAR optimized for NK‐cell biology that targets the antigen of interest, a novel high‐affinity 158V, noncleavable CD16 (hnCD16) Fc receptor, which has been modified to prevent its downregulation and to enhance its binding to tumor‐targeting antibodies, and an IL‐15 receptor fusion (IL‐15RF) that promotes enhanced NK‐cell activity [99]. CAR‐NK might represent a promising therapeutic option with all the benefits inherent to “off‐the‐shelf” therapies pending the clinical trial results.

### 8.2 Allogeneic alpha‐beta CAR‐T

Several allo‐CAR‐T clinical trials are employing conventional αβ T cells from healthy donors (Table 4). The following gene‐editing technologies have been used in generating allo‐CAR‐T.

#### 8.2.1 Transcription activator‐like effector nucleases

Transcription activator‐like effector nucleases (TALEN) technology is arguably the first gene editing technology used in the generation of universal allo‐CAR‐T for lymphoma patients in clinical trials. TALENs are transcription factors (hybrid molecules) linked to an endonuclease that can be engineered to cut specific DNA sequences [100]. Knocking out the TRAC gene locus is an attractive approach to disrupt the expression αβ TCR, thereby limiting the GVHD initiating potential of allo‐CAR‐T. By simultaneously electroporating TALENs that disrupted TCR and CD52 expression in the T cells, in the preclinical model, this methodology produced allo‐CAR‐T that did not induce GVHD and were resistant to anti‐CD52 monoclonal antibody used to eliminate host T cells [77]. The latter was employed as an immunosuppressive strategy to prevent recipient immune cell‐mediated rejection of CAR‐T. As shown in Table 4, Allogene 501 trial using this platform produced CR rates of 50% in patients DLBCL and FL with to date no reports of GVHD or frequent CRS or ICANS. These preliminary finds need confirmation with longer follow‐up and a larger sample size [101, 102].

#### 8.2.2 Meganuclease‐edited CARs

Meganucleases are a group of naturally occurring and highly specific restriction enzymes with gene‐editing potential. Precision BioSciences has developed a next‐generation meganuclease platform called “ARCUS” that can produce nucleases with customized activity and specificity [103, 104]. PBCAR0191 is an anti‐CD19 allo‐CAR‐T that disrupts TCR expression via CAR gene insertion in the TRAC locus [105] (Table 4), and is being tested in a Phase I/II study. At last follow‐up, 16 patients with aggressive NHL were treated [106]. The trial employed either standard or escalated doses (higher fludarabine and cyclophosphamide doses) of LD regimens. Overall, the study demonstrated a CR rate of 38%, but among four patients getting escalated LD, three achieved a CR. No episodes of grade ≥ 3 CRS or NAE were noted, but severe infections were more frequently seen with escalated LD.

#### 8.2.3 CRSIPR

CRISPR system is a simple, versatile, and precise gene‐editing tool with highly efficiency multiplex genomic‐editing capability [74, 79, 107–109]. Multiplex genome‐editing allows sequence‐specific gene delivery, resulting in a highly efficient 2‐in‐1 TCR knockout and CAR knock in for universal allo‐CAR‐T, with the advantages of significantly lower risks of insertional oncogenesis and TCR‐induced alloreactivity [103, 110]. Replacing the endogenous TCR with a CAR not only disrupts the TCR but also brings CAR under the regulatory control of the endogenous TCR promoter, leading to improved T‐cell function and potency [110]. Multiplex CRISPR/Cas9 has been used to generate allo‐universal CAR‐T deficient in TCR β chain, B2M, PD‐1, and CTLA‐4, which have been shown to maintain function in vitro and in vivo [74, 109, 111]. The  CRISPR Therapeutics’ CRISPR‐edited anti‐CD19 CAR‐T cell trial (CTX110) is ongoing and enrolling patients with B‐cell NHL (Table 4) [112].

#### 8.2.4 Epstein–Barr virus‐specific T cells

Epstein–Barr VSTs (EBVSTs) are virus‐specific and hence have limited TCR repertoire and therefore are less likely to mediate GVHD [113]. To prevent rejection, CD30 CAR can be introduced into “off‐the‐shelf” EBVSTs. CD30 CAR allows targeting CD30+ lymphomas and has proved safe and effective in clinical trials of auto‐CAR‐T [49]. A Phase I trial evaluating allo‐CD30 CAR EBVSTs (TT11X) therapy in patients with heavily pretreated CD30+ HL and NHL is ongoing (Table 4) [113].

### 8.3 Allogeneic gamma‐delta CAR‐T

Conventionally αβ T cells have been used for production of CAR‐T, however, γδ T cells may offer unique advantages over αβ T cells [114]. Despite the small number of γδ T cells present in peripheral blood, these cells can be expanded ex vivo to produce clinically significant yield for therapeutic effect [115]. Preclinical data have demonstrated γδ T‐cell expressing anti‐CD19 CAR have potent cytotoxicity toward CD19+ leukemia cell lines in vitro and in vivo [116]. γδ T cells can also recognize pathogen (including viral) stressed and transformed target cells in an HLA‐independent fashion and are activated in an allo‐setting without the concern of GVHD. γδ1 CAR T‐cell product targeting CD20 is now entering clinical trials for treatment of B‐cell malignancies (NCT04735471). The study is using selectively expanded γδ1 T cells from healthy donors that are engineered with a second‐generation CAR construct (4‐1BBz).

## 9 FUTURE DIRECTIONS

CAR‐T therapy is a revolutionary treatment for patients with R/R B‐cell lymphomas. Although the platform currently has multiple limitations as discussed, the future for CAR‐T appears promising with multiple strategies underway to increase efficacy and limit toxicity. The approval of CAR‐T in MCL and FL represents a significant advancement in the field, as these histologies have traditionally been considered incurable unlike LBCL. Whether CAR‐T can lead to a cure in these lymphomas remains to be proven, pending long‐term follow‐up data. Combining novel targeted agents with CAR‐T is another promising strategy. In preclinical MCL models, concurrent treatment with ibrutinib and CAR‐T resulted in improved responses and decreased toxicity [117]. This combination is rational as ibrutinib blocks inducible T‐cell kinase in addition to BTK and with resultant enhanced Th1‐type cellular immunity [118]; ongoing TRANSCEND‐004 clinical trial is evaluating this combination in patients with CLL (NCT03331198).

Expansion of CAR‐T to additional lymphoma histologies such as HL and TCL is expected, pending the results of ongoing trials; primary and secondary central nervous system lymphoma (PCNSL/SCNSL) represent orphan diseases with particularly poor outcomes for patients with R/R disease. ZUMA‐1 and JULIET excluded patients with CNSL; however, patients with SCNSL were allowed in TRANSCEND‐NHL‐001(5). Safety and efficacy were not significantly different from patients without CNS involvement; additional experience from the real‐world setting is also consistent with that of TRANSCEND‐NHL‐001(5) [119]. The three currently approved CD‐19‐directed CAR‐T in LBCL (i.e., axi‐cel, tisa‐cel, and liso‐cel) are being actively investigated in patients with PCNSL and SCNSL (NCT04608487) (NCT04134117) (NCT03484702); results are currently awaited. A recently published report from an ongoing Phase I trial (NCT02153580) has shown encouraging responses with manageable toxicities in a small subset of patients (*n* = 5) with PCNSL receiving CD19‐directed CAR‐T [120].

Current experience with CAR‐T therapy has clearly established that CAR‐T in lymphoma is here to stay. However, with the various platforms of CAR‐T therapy that are currently in development, there are multiple questions that emerge. Most importantly, whether a particular CAR design or cell type will be superior in terms of efficacy and safety. Allo‐CARs can potentially be the answer to the limitations currently experienced with auto‐CARs; however, the platform is associated with notable risks such as that of bidirectional alloreactivity and insertional oncogenesis among others. Second, how can these various therapeutic cell products be sequenced to allow for best long‐term outcomes in patients with lymphoma. Lastly, if the safety profile of a particular product would allow for widespread CAR‐T expansion in the community, particularly outpatient (OP) administration, 10% and 18% of patients received CAR‐T as an OP in TRANSCEND‐NHL‐001 and the ELARA trial, respectively. TRANSCEND‐OUTREACH‐007 (NCT03744676) is currently ongoing and is exploring the safety and efficacy of liso‐cel in the outpatient setting.

## AUTHOR CONTRIBUTIONS

MI and MH collected and analyzed the data, wrote the first draft, and approved the final version.

## CONFLICT OF INTEREST

Mehdi Hamadani reports Research Support/Funding: Takeda Pharmaceutical Company; Otsuka Pharmaceutical; Spectrum Pharmaceuticals; Astellas Pharma. Consultancy: Medimmune LLC; Janssen R & D; Incyte Corporation; ADC Therapeutics; Cellerant Therapeutics; Celgene Corporation; Pharmacyclics, Magenta Therapeutics, Omeros, AbGenomics, Verastem, TeneoBio. Speaker's Bureau: Sanofi Genzyme, AstraZeneca.
